# Outcomes of COVID-19 Among Hospitalized Health Care Workers in North America

**DOI:** 10.1001/jamanetworkopen.2020.35699

**Published:** 2021-01-28

**Authors:** Jeong Yun Yang, Michael D. Parkins, Andrew Canakis, Olga C. Aroniadis, Dhiraj Yadav, Rebekah E. Dixon, B. Joseph Elmunzer, Nauzer Forbes

**Affiliations:** 1 Department of Medicine, Icahn School of Medicine at Mount Sinai, New York, New York; 2 Division of Infectious Diseases, Department of Medicine, University of Calgary, Calgary, Alberta, Canada; 3 Section of Gastroenterology, Department of Medicine, Boston University Medical Center, Boston, Massachusetts; 4 Division of Gastroenterology, Stony Brook Hospital, Stony Brook, New York; 5 Division of Gastroenterology, Hepatology and Nutrition, University of Pittsburgh Medical Center, Pittsburgh, Pennsylvania; 6 Division of Gastroenterology and Hepatology, Medical University of South Carolina, Charleston; 7 Division of Gastroenterology and Hepatology, Department of Medicine, University of Calgary, Calgary, Alberta, Canada; 8 Department of Community Health Sciences, University of Calgary, Calgary, Alberta, Canada

## Abstract

**Importance:**

Although health care workers (HCWs) are at higher risk of acquiring coronavirus disease 2019 (COVID-19), it is unclear whether they are at risk of poorer outcomes.

**Objective:**

To evaluate the association between HCW status and outcomes among patients hospitalized with COVID-19.

**Design, Setting, and Participants:**

This retrospective, observational cohort study included consecutive adult patients hospitalized with a diagnosis of laboratory-confirmed COVID-19 across 36 North American centers from April 15 to June 5, 2020. Data were collected from 1992 patients. Data were analyzed from September 10 to October 1, 2020.

**Exposures:**

Data on patient baseline characteristics, comorbidities, presenting symptoms, treatments, and outcomes were collected, including HCW status.

**Main Outcomes and Measures:**

The primary outcome was a requirement for mechanical ventilation or death. Multivariable logistic regression was performed to yield adjusted odds ratios (AORs) and 95% CIs for the association between HCW status and COVID-19–related outcomes in a 3:1 propensity score–matched cohort, adjusting for residual confounding after matching.

**Results:**

In total, 1790 patients were included, comprising 127 HCWs and 1663 non-HCWs. After 3:1 propensity score matching, 122 HCWs were matched to 366 non-HCWs. Women comprised 71 (58.2%) of matched HCWs and 214 (58.5%) of matched non-HCWs. Matched HCWs had a mean (SD) age of 52 (13) years, whereas matched non-HCWs had a mean (SD) age of 57 (17) years. In the matched cohort, the odds of the primary outcome, mechanical ventilation or death, were not significantly different for HCWs compared with non-HCWs (AOR, 0.60; 95% CI, 0.34-1.04). The HCWs were less likely to require admission to an intensive care unit (AOR, 0.56; 95% CI, 0.34-0.92) and were also less likely to require an admission of 7 days or longer (AOR, 0.53; 95% CI, 0.34-0.83). There were no differences between matched HCWs and non-HCWs in terms of mechanical ventilation (AOR, 0.66; 95% CI, 0.37-1.17), death (AOR, 0.47; 95% CI, 0.18-1.27), or vasopressor requirements (AOR, 0.68; 95% CI, 0.37-1.24).

**Conclusions and Relevance:**

In this propensity score–matched multicenter cohort study, HCW status was not associated with poorer outcomes among hospitalized patients with COVID-19 and, in fact, was associated with a shorter length of hospitalization and decreased likelihood of intensive care unit admission. Further research is needed to elucidate the proportion of HCW infections acquired in the workplace and to assess whether HCW type is associated with outcomes.

## Key Points

**Question:**

Are health care workers (HCWs) at risk of worse outcomes associated with coronavirus disease 2019 (COVID-19) compared with the general population?

**Findings:**

This propensity-matched multicenter cohort study included 122 HCWs hospitalized with COVID-19 matched to 366 non-HCWs hospitalized with COVID-19. The odds of the primary outcome—mechanical ventilation or death—were not significantly different for HCWs compared with non-HCWs.

**Meaning:**

This study finds that HCW status is not associated with poorer outcomes among patients hospitalized with COVID-19.

## Teaser

This propensity score–matched multicenter cohort study assesses the association between health care worker status and outcomes among hospitalized patients with coronavirus disease 2019 (COVID-19) in North America.

## Introduction

Severe acute respiratory syndrome coronavirus 2 (SARS-CoV-2) was first recognized in North America in January 2020.^1^ Within weeks of this milestone, coronavirus disease 2019 (COVID-19), the clinical manifestation resulting from SARS-CoV-2 infection, was declared a pandemic by the World Health Organization.^2^ To date, there have been in excess of 75 million cases of COVID-19 worldwide, with more than 1.6 million associated deaths.^3^ The spectrum of disease and natural history of COVID-19 are both highly variable, with a relatively large number of asymptomatic or minimally symptomatic infections having been reported to date.^4^ Despite this, and even though a large proportion of infected patients recover fully, the possibility also exists of infection leading to prolonged hospitalization, severe disease course, or death.^5^ Although some risk factors for severe outcomes have been identified, they provide an incomplete understanding, and it remains relatively unclear what accounts for this large degree of observed variability in outcomes.^6^

One biologically plausible theory to explain discordant clinical outcomes involves the concept of the infectious dose to which each patient is exposed, often expressed as the 50% infectious dose. Broadly speaking, the 50% infectious dose refers to the minimum number of viral particles required to successfully infect 50% of a reference population via a given route. Its determination has been crucial in developing epidemiologic models and treatments for other better understood viruses.^7,8^ The association between inoculum and mortality has been well documented in many respiratory viruses, including influenza^9^ and measles.^10^ Although the 50% infectious dose has been calculated for other coronaviruses, including SARS-CoV-1,^11^ and independently associated with outcomes,^12^ this value remains unknown to date for COVID-19.

Health care workers (HCWs) are at increased risk of acquiring COVID-19.^13,14^ Less than 1 month after COVID-19 was declared a pandemic, nearly 10 000 HCWs in the United States alone were diagnosed as having the virus.^15^ The rate of infection among HCWs varies widely based on numerous factors, including, among others, geographic region and local infection control policies.^5,16,17^ However, it also stands to reason that HCWs, because of their repeated and prolonged interactions with patients with the most severe COVID-19, could also be exposed to higher infectious doses at the time of infection compared with the general population, but this postulation remains unproven. Conversely, animal and human studies have shown that both asymptomatic and mild infections are more likely than severe infections with proper face coverings,^18,19^ which are commonly used as part of routine personal protective equipment (PPE) practices among HCWs. In addition, HCWs as an overall cohort may be healthier, younger, or both compared with non-HCWs.

Thus, it remains unclear whether HCWs are at higher, lower, or similar risk of severe COVID-19–related outcomes, including intensive care unit (ICU) admission and death. No clear differences in COVID-19–related outcomes have previously been shown for HCWs compared with the general population;^15^ however, to our knowledge, no studies have focused specifically on the outcomes of HCWs hospitalized with COVID-19. Therefore, we aimed to assess the potential differences in COVID-19–related outcomes between HCWs and matched non-HCW controls.

## Methods

### Study Design and Setting

This study followed the Strengthening the Reporting of Observational Studies in Epidemiology (STROBE) reporting guideline for reporting observational studies.^20^ This study assessed data from a cohort of individuals hospitalized with COVID-19 from 36 North American medical centers.^21^ Site-specific institutional ethics review board approval was obtained prior to patient identification, data collection, or deidentified data transfer. A waiver of informed consent was obtained from each center’s ethics review board because no intervention was performed as part of the study and because of the retrospective nature of the study. No one received compensation or was offered any incentive for participating in this study.

### Study Patients

Patients at least 18 years of age were eligible for study inclusion if they (1) had received a confirmed diagnosis of COVID-19 as per local standards of testing, including by antigen-based or nucleic acid–based tests, and (2) were hospitalized for any length of time. All attempts were made to consecutively enroll patients meeting eligibility criteria at each site, starting from the first inpatient with COVID-19. Several site-dependent methods were used to identify eligible patients, including (but not limited to) searches of public health repositories, acquisition of hospital-based service lists, and queries of data registries.

### Data Collection and Study Variables

Data were collected between April 15 and June 5, 2020. A secure electronic data collection form was designed to collect all relevant deidentified variables. Specific data elements were acquired via full review of all available electronic medical records by site-specific study personnel; no in-person patient or collateral interviews took place. Broadly, collected data fell into the following categories: patient demographic characteristics and comorbidities (including self-reported, caretaker-reported, or clinician-reported HCW status), medications (including COVID-19–specific treatments), presenting signs or symptoms, investigations, and relevant outcomes. The HCW status was defined as any role involving direct patient care as part of one’s daily responsibilities (including but not limited to physicians and nurses). The HCW status was only concluded if it was clear from the electronic medical record review whether a patient did or did not definitively meet the definition; otherwise, patients were classified as unknown with respect to HCW status. Given our retrospective approach, confirmation of the location where infection was acquired (nosocomial vs other) was not possible. All data were collected from symptom onset until hospital discharge, death, or the end of the study period. The full data collection form is provided in the eAppendix in the Supplement. All data were reviewed by a central data manager to ensure accuracy and consistency. Any missing, duplicate, or erroneous data or any other general data concerns were resolved by querying the site from which they arose.

### Outcomes

The primary outcome was a composite end point of mechanical ventilation or death. Secondary outcomes included individual components of the primary outcome, ICU admission, any requirement for vasopressor support, and hospital length of stay.

### Statistical Analysis

Study variables are reported using descriptive statistics and were compared using the *t* test for measured variables and the χ^2^ test for categorical variables. Comparative results are reported along with respective standardized mean differences (SMDs), with SMDs of 0.10 or higher considered statistically significant. To control for predetermined clinically relevant potential confounders, we then created individual propensity scores for each patient in the cohort. The covariates used to create those scores are given in full in the eTable in the Supplement and included sex, age, race, ethnicity, history of cigarette smoking and alcohol use, relevant comorbidities, presenting symptoms, and COVID-19 treatments. Propensity score matching in a 3:1 ratio was then performed to match patients with confirmed non-HCW status and those with confirmed HCW status, excluding patients with unknown HCW status. Caliper matching without replacement was used, with an a priori caliper width set at 0.25 times the SD of the propensity score.^22^

Any covariates whose SMDs were 0.10 or higher even after matching were included in a subsequent multivariable model. Multivariable conditional logistic regression was then performed to account for expected correlations between matched sets, with results expressed using adjusted odds ratios (AORs) along with respective 95% CIs and *P* values (all tests 2-sided, with *P* < .05 considered statistically significant). All data analyses were performed from September 10 to October 1, 2020, using SAS, version 9.4 (SAS Institute Inc). Propensity score matching was performed using methods outlined by Gant and Crowland.^23^

## Results

Data were collected from 1992 patients hospitalized with a diagnosis of COVID-19 across 36 North American centers. After excluding 202 patients with an uncertain HCW status, 1790 patients were included in the final study cohort, comprising 127 HCWs and 1663 non-HCWs. Clinically relevant characteristics of the study cohort are provided in Table 1. The HCWs were significantly younger than the non-HCWs, with a mean (SD) age of 53 (14) years vs 63 (17) years (SMD, 0.62). A significantly larger proportion of HCWs were women compared with non-HCWs (76 [59.8%] vs 696 [41.9%]; SMD, 0.37). A larger proportion of HCWs had never smoked compared with non-HCWs (91 [71.7%] vs 969 [58.3%]; SMD, 0.28). The non-HCWs had a higher degree of comorbidities than HCWs per the Charlson Comorbidity Index (CCI), with mean (SD) CCI scores of 1.6 (1.8) vs 0.9 (1.4). Comparisons of presenting symptoms, COVID-19–specific treatments, and outcomes are given in Table 1.

**Table 1.** Patient Demographic and Clinical Characteristics and Outcomes by HCW Status for Overall and Propensity Score–Matched Cohorts^a^

Characteristic	Overall cohort (n = 1790)			3:1 Propensity-matched cohort (n = 488)		
	No. (%)		SMD	No. (%)		SMD
	HCWs (n = 127)	Non-HCWs (n = 1663)		HCWs (n = 122)	Non-HCWs (n = 366)	
Female sex	76 (59.8)	696 (41.9)	0.37	71 (58.2)	214 (58.5)	0.01
Age, mean (SD), y	53 (14)	63 (17)	0.62	52 (13)	57 (17)	0.29^b^
Race						
White	44 (34.7)	629 (37.9)	0.07	41 (33.6)	114 (31.2)	0.05
Black/African American	51 (40.2)	702 (42.2)	0.04	49 (40.2)	150 (41.0)	0.02
Other/unknown	32 (25.2)	332 (19.9)	0.13	32 (26.2)	102 (27.9)	0.04
Ethnicity						
Hispanic/Latin(x)	8 (6.30)	234 (14.1)	0.26	8 (6.6)	31 (8.5)	0.07
Not Hispanic/Latin(x)	100 (78.7)	1299 (78.1)	0.02	95 (77.9)	285 (77.9)	0.02
Unknown	19 (15.0)	130 (7.8)	0.23	19 (15.6)	50 (13.7)	0.05
History of cigarette smoking						
Current	7 (5.5)	101 (6.1)	0.02	7 (5.7)	18 (4.9)	0.04
Ex-smoker	23 (18.1)	491 (29.5)	0.27	23 (18.9)	52 (14.2)	0.13^b^
Nonsmoker	91 (71.7)	969 (58.3)	0.28	86 (70.5)	282 (77.1)	0.15^b^
Unknown	6 (4.7)	102 (6.1)	0.06	6 (4.9)	14 (3.8)	0.05
Alcohol use						
Current	7 (5.5)	140 (8.4)	0.11	7 (5.7)	19 (5.2)	0.02
Prior	1 (0.8)	93 (5.6)	0.28	1 (0.8)	2 (0.6)	0.03
None	105 (82.7)	1250 (75.2)	0.18	100 (82.0)	313 (85.5)	0.10^b^
Unknown	14 (11.0)	180 (10.8)	0.01	14 (11.5)	32 (8.7)	0.09
Obesity	67 (52.8)	788 (47.4)	0.11	63 (51.6)	204 (55.7)	0.08
BMI, mean (SD)	31.9 (8.6)	31.3 (8.6)	0.08	31.8 (8.7)	32.8 (9.0)	0.13^b^
CCI, mean (SD)	0.9 (1.4)	1.6 (1.8)	0.50	0.9 (1.4)	1.1 (1.6)	0.14^b^
Diabetes	28 (22.1)	605 (36.4)	0.32	28 (23.0)	86 (23.5)	0.01
Hypertension	60 (47.2)	1055 (63.4)	0.33	59 (48.4)	177 (48.4)	0.00
Cardiac disease	16 (12.6)	381 (22.9)	0.27	16 (13.1)	53 (14.5)	0.04
Pulmonary disease	36 (28.4)	343 (20.6)	0.18	33 (27.1)	101 (26.0)	0.02
Active/current malignant neoplasm	6 (4.72)	110 (6.6)	0.08	6 (4.9)	17 (4.6)	0.01
Immunocompromised status	16 (12.6)	218 (13.1)	0.02	16 (13.1)	45 (12.3)	0.02
Luminal gastrointestinal tract disease	5 (3.9)	73 (4.4)	0.02	5 (4.1)	13 (3.6)	0.03
Pancreaticobiliary disease	4 (3.2)	43 (2.6)	0.03	3 (2.5)	8 (2.2)	0.02
Chronic liver disease	5 (3.9)	47 (2.8)	0.06	4 (3.3)	10 (2.7)	0.03
Presenting symptoms						
Fever	103 (81.1)	1271 (76.4)	0.11	98 (80.3)	301 (82.2)	0.05
Cough	106 (83.5)	1220 (73.4)	0.25	101 (82.7)	315 (86.1)	0.09
Dyspnea	99 (78.0)	1150 (69.2)	0.20	95 (77.9)	289 (79.0)	0.03
Fatigue/subjective weakness	56 (44.1)	705 (42.4)	0.03	54 (44.3)	163 (44.5)	0.01
Myalgia	54 (42.5)	453 (27.2)	0.32	51 (41.8)	150 (41.0)	0.02
Diarrhea	58 (45.7)	543 (32.7)	0.27	55 (45.1)	165 (45.1)	0.00
Nausea	49 (38.6)	422 (25.4)	0.29	44 (36.1)	137 (37.4)	0.03
Vomiting	29 (22.8)	255 (15.3)	0.19	27 (22.1)	79 (21.6)	0.01
Abdominal pain	14 (11.0)	186 (11.2)	0.01	13 (10.7)	45 (12.3)	0.05
COVID-19 treatment						
Hydroxychloroquine/ chloroquine	60 (47.2)	865 (52.0)	0.10	59 (48.4)	169 (46.2)	0.04
Remdesivir	8 (6.3)	91 (5.5)	0.04	8 (6.6)	22 (6.0)	0.02
Convalescent plasma	1 (0.8)	35 (2.1)	0.11	1 (0.8)	2 (0.6)	0.03
Glucocorticoids	14 (11.0)	204 (12.3)	0.04	14 (11.5)	43 (11.8)	0.01
Tocilizumab	4 (3.2)	88 (5.3)	0.11	4 (3.3)	14 (3.8)	0.03
Outcome						
Death	7 (5.5)	327 (19.7)	0.41	7 (5.7)	45 (12.3)	0.23
ICU admission	33 (26.0)	753 (45.3)	0.31	32 (26.2)	139 (38.0)	0.25
Mechanical ventilation	24 (18.9)	540 (32.5)	0.34	23 (18.9)	94 (25.7)	0.16
Vasopressor support	18 (14.2)	461 (27.7)	0.44	18 (14.8)	84 (23.0)	0.21
Hospital length of stay, median (IQR), d	6 (3-12)	9 (5-18)	0.44	6 (3-12)	8 (4.0-14)	0.31

Abbreviations: BMI, body mass index (calculated as weight in kilograms divided by height in meters squared); CCI, Charlson Comorbidity Index; COVID-19, coronavirus disease 2019; HCW, health care worker; ICU, intensive care unit; IQR, interquartile range; SMD, standardized mean difference.

^a^ A full list of covariates used to calculate propensity scores is provided in the eTable in the Supplement.

^b^ An SMD of 0.10 or higher in a nonoutcome parameter after 3:1 propensity score matching.

After 3:1 propensity score matching, there remained 122 HCWs matched to 366 non-HCWs (a total of 488 patients). The match was successful in achieving a greater balance across covariates, shown by the residual SMDs in Table 1. Women comprised 71 (58.2%) of matched HCWs and 214 (58.5%) of matched non-HCWs. Matched HCWs had a mean (SD) age of 52 (13) years, whereas matched non-HCWs had a mean (SD) age of 57 (17) years. Age, smoking and alcohol use, body mass index, and CCI scores all had persistent SMDs of 0.10 or higher even after matching. With the use of a conditional multivariable logistic regression model including those parameters as covariates, the odds of mechanical ventilation or death (the primary outcome) were not significantly different for HCWs compared with non-HCWs (AOR, 0.60; 95% CI, 0.34-1.04). However, HCWs were significantly less likely to require ICU admission (AOR, 0.56; 95% CI, 0.34-0.92) and were also significantly less likely to require an admission of 7 days or longer (AOR, 0.53; 95% CI, 0.34-0.83). There were no differences between matched groups of mechanical ventilation (AOR, 0.66; 95% CI, 0.37-1.17) or death (AOR, 0.47; 95% CI, 0.18-1.27) (as individual outcomes) or requirement of vasopressor support (AOR, 0.68; 95% CI, 0.37-1.24). Table 2 gives the propensity score–matched and propensity score–unmatched conditional logistic regression results for all primary and secondary outcomes.

**Table 2.** Primary and Secondary Outcomes for Patients With HCW Status (vs Patients With Non-HCW Status) Assessed With Conditional Multivariable Logistic Regression^a^

Outcome	Full cohort (n = 1790)		3:1 Propensity matched cohort (n = 488)	
	AOR (95% CI)	*P* value	AOR (95% CI)	*P* value
Mechanical ventilation or death (primary outcome)	0.45 (0.27-0.73)	.001	0.60 (0.34-1.04)	.07
Death	0.35 (0.14-0.88)	.03	0.47 (0.18-1.27)	.14
ICU admission	0.41 (0.26-0.64)	<.001	0.56 (0.34-0.92)	.02^b^
Mechanical ventilation	0.47 (0.28-0.78)	.003	0.66 (0.37-1.17)	.15
Vasopressor support	0.49 (0.28-0.85)	.01	0.68 (0.37-1.24)	.21
Hospital length of stay ≥7 d	0.49 (0.33-0.73)	<.001	0.53 (0.34-0.83)	.006^b^

Abbreviations: AOR, adjusted odds ratio; HCW, health care worker; ICU, intensive care unit.

^a^ Conditional logistic regression models included age, body mass index, Charlson Comorbidity Index, smoking status (current or ex-smoker vs nonsmoker), and alcohol use (current or prior use vs none) as covariates.

^b^ Statistically significant association after 3:1 propensity score matching.

## Discussion

In this study, hospitalized individuals with confirmed HCW status did not experience worse COVID-19–related outcomes compared with a matched non-HCW cohort. In fact, HCW status was modestly but significantly associated with a lack of requirement for ICU admission and with a shorter overall length of hospitalization. These findings are important given the expected ongoing burden of disease during the COVID-19 pandemic.

Our results are consistent with those of other relevant investigations to date.^15,24^ However, it is noteworthy that only 1 prior study addressing COVID-19–related outcomes among HCWs has focused on hospitalized patients.^24^ In an unmatched study from Wuhan, China, HCWs were observed to die of COVID-19 at a rate much lower than the general population, despite being infected at higher rates.^25^ The HCWs in the United States and in the United Kingdom have also been shown to be disproportionately affected, with at least a 3-fold increased risk of a COVID-19 diagnosis in a recent large study.^14^ This observation is common to other infectious outbreaks, including SARS-CoV-1.^26^ However, while the risk of COVID-19 is higher among HCWs relative to the general population, that risk is highly variable, depending on the timing within the pandemic and the intensity and duration of the exposure.^14^ It appears that nurses are more likely than physicians to acquire COVID-19.^25^ This is probably not surprising given that it is well established that nurses, on average, spend the most time relative to other groups with individual patients, thereby increasing their total risk of exposure as well as their potential infectious dose.^27,28^ Whether nurses might experience a disproportionate burden of disease relative to physicians with COVID-19 (with regard to morbidity and mortality) is not yet known.

The PPE practices and availability are important risk factors associated with nosocomial infection, and both vary from site to site.^29^ In a large prospective study, both reuse of PPE and inadequate PPE were independently associated with an increased risk of acquiring COVID-19 among HCWs.^14^ However, that study was conducted prior to the more evidence-based disinfection and reuse protocols currently being instituted.^30,31^ Our results showing equivalent or slightly better outcomes among HCWs with infection may potentially be explained by meticulous PPE use following approved protocols in workplace settings, and compliance with these protocols would be expected to improve over time. However, one must also acknowledge the potential for a healthy worker effect among HCWs, a phenomenon in which the index population (HCWs in our case) may be inappropriate for comparison with the general population simply because they are sufficiently healthy to be employed.^32^ Indeed, the HCWs in our population were younger, had lower CCI scores, and were more likely to be nonsmokers, all reasons why propensity score matching was essential to balance these potential confounders. However, other unmeasured confounders likely also were associated with important differences between our 2 study populations, including, as a possibility, the degree of compliance with social distancing protocols observed by HCWs vs the general population. The possibility also exists that HCWs were able to receive more prompt diagnoses or treatment owing to their preexisting connections to the medical community.

Our findings demonstrate that HCW status is not specifically associated with poorer outcomes among hospitalized patients. However, one must use caution when applying these results to nonhospitalized HCWs with COVID-19, for whom several unique patient care considerations apply. Specifically, it is critical to recognize the physical, psychological, social, and practical burdens of this disease on HCWs, who collectively represent a disproportionately affected population. In a meta-analysis performed in April 2020, anxiety was prevalent in more than 23% of HCWs, with insomnia being prevalent in 39%.^33^ Given that the pandemic has endured for months since that study was carried out, these values could well underestimate the current situation. To add to this baseline state of unrest, HCWs with COVID-19 may be susceptible to experiencing additional negative emotions, such as frustration or helplessness^34^ in addition to shame or stigmatization by colleagues. Workplaces should ensure that mechanisms are in place to both identify and support at-risk HCWs, including potential routine screening of personnel to evaluate risk factors for (or symptoms of) depression, anxiety, or high stress.^35^

### Limitations

One notable limitation of our study is the inability to distinguish where HCWs were exposed and subsequently developed their infection. Many HCWs with COVID-19 appear to have acquired infection from the community or from travel, as opposed to through workplace exposures. Indeed, detailed epidemiological investigations associated with whole-genome sequencing data suggest that the majority of HCW infections are not acquired within hospitals.^36^ Given the inability within large data sets (including our own) to distinguish where COVID-19 exposures occurred among HCWs, alternative approaches are necessary to study how exposure intensity in HCWs may affect outcomes, ideally prospectively selecting only HCWs that were confirmed to be exposed through their workplace. Several other limitations are inherent to our retrospective study design, including reliance on medical record reviews as opposed to direct patient interviews. For instance, we were unable in either group to adjust for the extent of out-of-hospital exposure, including from COVID-19–positive household contacts, which is an important omission. Other limitations include the relatively small size of our final matched cohort, but as mentioned, this was counterbalanced by the rigor of the approach we used to address this specific question. Of note, propensity score matching is also not without limitations, given its inability to control for unmeasured confounders, despite the high number and robust granularity of known potential confounders incorporated into our models. Given both our methods and ultimate sample size, we were also unable to stratify outcomes by type of HCW, which, as we discussed, is an important parameter because it may be associated with infectious dose. Furthermore, the restriction of our cohort to hospitalized patients may also lead to potential limitations in not being able to analyze factors such as differential rates of testing and admission in HCWs relative to the general population. In addition, our study included inpatients across North America, and therefore caution should be exercised in generalizing its results to outpatients or to other regions given the high degree of geographic variability associated with all aspects of the pandemic. Finally, the burden of death and other severe outcomes in hospitalized patients with COVID-19 was relatively high in our study, which could be explained by data collection having been carried out in the early stages of the pandemic. In these early stages, medical expertise in treating the disease was not as developed as it is today; for example, understanding of and guidance on the management of critically ill patients with COVID-19 have since evolved, including regarding endotracheal intubation and mechanical ventilation.^37^ This also somewhat limits the generalizability of our findings.

## Conclusions

In summary, we found that HCW status did not appear to be associated with poorer outcomes among hospitalized patients with COVID-19 but was associated with lower ICU admission rates and a shorter overall hospital length of stay. Proper PPE practices are critical to the ongoing protection of frontline health care staff. Further research is needed to elucidate the proportion of HCW infections acquired in the workplace and to assess whether HCW type is associated with outcomes.

## Notes

Supplement. **eAppendix.** Data Collection Form **eTable.** Covariates Used to Calculate Propensity Scores
