# Supplementary material for: Outcomes of COVID-19 Among Hospitalized Health Care Workers in North America
Source: JAMA Netw Open. 2021 Jan 28;4(1):e2035699. doi: 10.1001/jamanetworkopen.2020.35699 (PMC7844592; doi:10.1001/jamanetworkopen.2020.35699)
Supplement: Supplement. — eAppendix. Data Collection Form eTable. Covariates Used to Calculate Propensity Scores [file jamanetwopen-e2035699-s001.pdf]

Supplementary Online Content

Yang JY, Parkins MD, Canakis A, et al; DMC-19 Study Group; North American Alliance for the Study of Digestive Manifestations of COVID-19. Outcomes of COVID-19 among hospitalized health care workers in North America. *JAMA Netw Open*. 2021;4(1):e2035699. doi:10.1001/jamanetworkopen.2020.35699

eAppendix. Data Collection Form

eTable. Covariates Used to Calculate Propensity Scores

This supplementary material has been provided by the authors to give readers additional information about their work.

© 2021 Yang JY et al. *JAMA Network Open*.

eAppendix. Data Collection Form

The electronic data collection form contained 216 items and was divided into 5 sections: 1) patient characteristics; 2) COVID-19 parameters; 3) symptomatology; 4) imaging and endoscopy; and 5) laboratory data and other hepatic considerations. The form was developed by a core group of investigators and was refined and finalized after a 72-hour period of public comment.

DMC19 Registry Instructions 4_17_20

1) Please complete the below data collection form (DCF) in REDCap at the **time of discharge or death**. Data will appear in the DMC19 database once entry and verification are complete.

2) We aim to capture **inpatients** with a confirmed COVID-19 diagnosis, **regardless of whether they have digestive manifestations**. After prevalence is defined in hospitalized patients, and as the numbers grow, we may focus on patients who are known to have GI manifestations and/or include outpatients.

3) Please make all efforts to collect data on the **first 50-100 consecutive** patients at your hospital or health system.

4) Eligible patients can and should be identified by any means necessary, which may include, but is not limited to, institutional laboratory records, data warehouse queries, electronic health record research subject identification tools/dashboards, and discussions with the infectious disease or critical care services, etc. You may elect to use the emergency ICD-10 code of U07.1 – 2019-nCov acute respiratory disease – to help identify eligible patients.

5) Please **triple check** data for accuracy before submission. Although we are performing central data monitoring, we cannot verify incoming data against source documents, nor are we performing on-site monitoring visits. Therefore, the overall quality of the data is assured primarily at the site level.

6) Along the lines of #5, coordinators should confer with a clinician during data collection to ensure that clinical context is accounted for as much as possible in the interpretation of questions that involve an element of subjectivity.

7) All data fields should have affirmative, negative, and unknown options. Therefore, missing data will be assumed to be inadvertent and this will generate a query.

8) Please maintain a secure key at your site that allows patient identification on the basis of subject ID#. This may be used in the future for to collect data pertaining to long-term outcomes.

**Subject ID #**	
**Institution**	
**Email address** (of individual entering data)	

**Patient characteristics**	
Age (years)	
Sex	☐Male

© 2021 Yang JY et al. *JAMA Network Open*.

	☐Female<br>- Pregnant<br>- Not pregnant<br>- Unknown
Race (check all that apply)	☐American Indian or Alaska Native<br>☐Asian<br>☐Black or African American<br>☐Native Hawaiian or other Pacific Islander<br>☐White<br>☐Unknown
Ethnicity	☐Hispanic or Latino<br>☐Not Hispanic or Latino<br>☐Unknown
Body mass index at presentation (kg/m^2): available?	☐Yes<br>☐Cannot calculate, but obesity documented<br>☐Cannot calculate
Body mass index at presentation (kg/m^2)	
Is the patient a health care worker?	☐Yes<br>☐No<br>☐Unknown
Cigarette smoking status	☐Current smoker<br>☐Ex-smoker<br>☐Non-smoker<br>☐Unknown
Vaping status	☐Current vaping<br>☐Prior vaping<br>☐Does not vape<br>☐Unknown
Alcoholism	☐Yes, current<br>☐Prior<br>☐No<br>☐Unknown
Cannabis use	☐Current user<br>☐Prior user<br>☐No<br>☐Unknown
Illicit drug use	☐Current user<br>☐Prior user<br>☐No<br>☐Unknown
Comorbidities (select all that apply)	☐Hypertension<br>☐Coronary artery disease/prior or current myocardial infarction (MI)<br>☐Congestive heart failure (CHF)<br>☐Chronic pulmonary obstructive disease (COPD)<br>☐Asthma

© 2021 Yang JY et al. *JAMA Network Open*.

	☐Obstructive sleep apnea (OSA)<br>☐Interstitial lung disease (ILD)/pulmonary fibrosis<br>☐Peripheral vascular disease (PVD)<br>☐Prior or current cerebrovascular accident (CVA) or transient ischemic attack (TIA)<br>☐Dementia<br>☐Collagen vascular/rheumatologic disease<br>☐Chronic liver disease<br>- Alcoholic liver disease<br>- Non-alcoholic fatty liver disease (NAFLD)<br>- Hepatitis C virus<br>- Hepatitis B virus<br>- Other, specify<br>☐Cirrhosis<br>If yes, MELD score prior to COVID-19 illness<br>☐Diabetes mellitus, uncomplicated<br>☐Diabetes mellitus with end-organ damage<br>☐Moderate to severe kidney disease (creatinine >3 mg/dL prior to admission, end stage renal disease [ESRD], dialysis)<br>☐Active/Current malignancy, excluding non-melanoma skin cancer<br>If yes, specify<br>☐Prior malignancy<br>☐Human immunodeficiency virus (HIV)/Acquired Immunodeficiency Syndrome (AIDS)<br>☐Solid organ transplant recipient<br>☐Bone marrow transplant recipient<br>☐Irritable bowel syndrome<br>☐Chronic diarrhea<br>☐Chronic constipation<br>☐Celiac disease<br>☐Prior biliary disease, including cholelithiasis, cholecystitis, choledocholithiasis or cholangitis<br>☐Prior pancreatitis<br>If yes: ☐Acute pancreatitis<br>☐Recurrent acute pancreatitis<br>☐Chronic pancreatitis<br>☐Unknown<br>☐Inflammatory bowel disease<br>☐None<br>☐Other, not fitting into an above category
If other, specify	
Recent (within 6 months) or current (at admission) immunosuppression or chemotherapy	☐Yes<br>☐No<br>☐Unknown

© 2021 Yang JY et al. *JAMA Network Open*.

If yes, specify	(medication, dose, route, duration)
Recent (within 1 month of admission) or current (at admission) ACE inhibitor use	☐Yes<br>☐No<br>☐Unknown
Recent (within 1 month of admission) or current (at admission) angiotensin receptor blocker (ARB) use	☐Yes<br>☐No<br>☐Unknown
Recent (within 1 month of admission) or current (at admission) NSAIDs use	☐Yes<br>☐No<br>☐Unknown
Recent (within 1 month of admission) or current (at admission) antibiotic use	☐Yes<br>☐No<br>☐Unknown
Recent (within 1 month of admission) or current (at admission) PPI use	☐Yes<br>☐No<br>☐Unknown
Recent (within 1 month of admission) or current (at admission) H2 blocker use	☐Yes<br>☐No<br>☐Unknown

**COVID-19 parameters**	
History of known contact with COVID-19 positive individual(s)	☐Yes<br>☐No<br>☐Unknown
Highest level of care	☐Inpatient ward<br>☐Intensive care unit (ICU)
Duration of symptoms prior to first seeking medical attention (days)	
Duration of symptoms prior to hospitalization (days)	
Duration of hospitalization (days)	
If admitted to ICU, duration of ICU stay (days)	
Required mechanical ventilation	☐Yes<br>☐No
Required extracorporeal membrane oxygenation (ECMO)	☐Yes<br>☐No
Required vasopressor support	☐Yes<br>☐No
Final discharge disposition	☐Recovered (or almost recovered)<br>☐Discharged to rehab or nursing facility<br>☐Deceased
COVID-specific treatments (select all that apply)	☐Remdesivir<br>☐Hydroxychloroquine<br>☐Chloroquine

© 2021 Yang JY et al. *JAMA Network Open*.

	☐Azithromycin<br>☐Glucocorticoids<br>☐Interferon alpha<br>☐Intravenous immunoglobulin (IVIG)<br>☐Lopinavir/ritonavir<br>☐Oseltamivir<br>☐Tocilizumab<br>☐Convalescent plasma<br>☐None<br>☐Other
If other, specify	(medication, dose, route, duration)

**Symptomatology**	
Respiratory or systemic symptoms (select all that apply)	☐Fever (subjective or objective)<br>☐Chills/rigors<br>☐Fatigue or subjective weakness<br>☐Myalgia<br>☐Sore throat<br>☐Rhinorrhea (runny nose)<br>☐Cough<br>☐Sneezing<br>☐Sputum production<br>☐Shortness of breath<br>- At rest<br>- On exertion<br>- Not specified<br>☐Chest tightness or pain<br>☐Headache<br>☐Confusion or altered mental status<br>☐Loss of smell<br>☐Loss of taste<br>☐None<br>☐Other
If other, specify	
Gastrointestinal symptoms or signs (select all that apply)	☐Anorexia<br>☐Nausea<br>☐Vomiting<br>☐Abdominal pain (including cramps)<br>-Diffuse<br>-Periumbilical<br>-Epigastric<br>-Right upper quadrant (RUQ)<br>-Left upper quadrant (LUQ)<br>-Right lower quadrant (RLQ)<br>-Left lower quadrant (LLQ)

© 2021 Yang JY et al. *JAMA Network Open*.

	-Not specified<br>☐Diarrhea<br>-Maximum number of bowel movements in a 24 hour period ☐Not documented<br>☐Bloody diarrhea<br>-Maximum number of bowel movements in a 24 hour period ☐Not documented<br>☐Hematemesis<br>☐Melena<br>☐Hematochezia (inc. bright red blood per rectum)<br>☐Dysphagia<br>☐Odynophagia<br>☐Constipation<br>☐Hiccups<br>☐Jaundice<br>☐None<br>☐Other
If other, specify	
Timing of gastrointestinal symptoms (if any) relative to respiratory/systemic symptoms (if any)	☐GI symptom(s) **preceded** other symptoms<br>☐GI symptom(s) **followed** other symptoms<br>☐GI symptom(s) **came on concurrently** with other symptoms<br>☐GI symptoms were the **only** manifestation<br>☐Can't tell from medical records review
Duration of gastrointestinal symptoms	☐GI symptom(s) were present for a short portion (<25% of the duration) of the entire COVID-19 illness<br>☐GI symptom(s) were present for significant portion (25-75% of the duration) of the entire COVID-19 illness<br>☐ GI symptom(s) were present for the entire/almost entire COVID-19 illness<br>☐Can't tell from medical records review
Did gastrointestinal symptoms remain after resolution of other COVID-19 symptoms?	☐Yes<br>☐No<br>☐Unknown
If yes, how long (days)	
Prominence of gastrointestinal symptoms	☐GI symptoms were less prominent than other symptoms related to COVID-19<br>☐GI symptoms were equally prominent to other symptoms related to COVID-19<br>☐GI symptoms were more prominent than other symptoms related to COVID-19<br>☐Can't tell from medical records review
Were digestive manifestations (gastrointestinal or hepatic) specifically	☐Yes, GI symptoms<br>☐Yes, LFT abnormalities

© 2021 Yang JY et al. *JAMA Network Open*.

addressed in the Assessment/Plan section of any progress notes for **3 or more days** during the hospitalization?	☐Yes, both<br>☐No
Did the gastroenterology or hepatology service consult on the patient during the hospitalization, as evidence by consult notes?	☐Yes, GI (including pancreaticobiliary)<br>☐Yes, hepatology/liver<br>☐Yes, both<br>☐No
Were stool studies other than FOBT (fecal occult blood test) obtained during the hospitalization?	☐Yes<br>☐No
Gastrointestinal diagnoses established shortly before, during, or shortly after COVID-19 illness (select all that apply)	☐Esophagitis/esophageal ulcers<br>☐Gastritis<br>☐Peptic ulcer disease (stomach or duodenum)<br>☐New enteritis<br>☐New colitis<br>☐Biliary disease, including cholelithiasis, cholecystitis, choledocholithiasis or cholangitis<br>☐Hepatitis<br>☐Pancreatitis<br>☐None<br>☐Other
If other, specify	
Was COVID-specific treatment prescribed specifically for digestive manifestations?	☐Yes<br>☐No<br>☐Unknown

**Imaging and endoscopy**	
Was abdominal computed tomography (CT) **performed and abnormal** shortly before, during, or shortly after COVID-19 illness?	☐Yes<br>☐No<br>☐Unknown
If yes, list abnormal findings in the impression section of the most concerning CT report	
Was abdominal magnetic resonance imaging (MRI) **performed and abnormal** shortly before, during, or shortly after COVID-19 illness?	☐Yes<br>☐No<br>☐Unknown
If yes, list abnormal findings in the impression section of the most concerning MRI report	
Was abdominal ultrasound (US) **performed and abnormal** shortly before, during, or shortly after COVID-19 illness?	☐Yes<br>☐No<br>☐Unknown

© 2021 Yang JY et al. *JAMA Network Open*.

If yes, list abnormal findings in the impression section of the most concerning US report	
Was endoscopy performed during COVID-19 illness? (select all that apply)	☐Yes – upper endoscopy (EGD)<br>☐Yes – colonoscopy or flexible sigmoidoscopy<br>☐Yes – enteroscopy (balloon or push)<br>☐Yes – capsule endoscopy<br>☐Yes – ERCP<br>☐Yes – endoscopic ultrasound (EUS)<br>☐No<br>☐Unknown
If performed, how many total endoscopic sessions did the patient undergo? (EGD+colonoscopy or EUS+ERCP at the same time is considered 1 session)	
If performed, did the patient require respiratory support **BEFORE** their first procedure? (select all the apply)	☐Yes – nasal cannula<br>☐Yes – high-flow oxygen<br>☐Yes – non-invasive positive pressure ventilation<br>☐Yes – mechanical ventilation<br>☐Yes – mechanical ventilation, proned<br>☐Yes – extracorporeal membrane oxygenation (ECMO)<br>☐No<br>☐Unknown
If performed, what kind of anesthesia did the patient receive **DURING** their first procedure? (select all that apply)	☐Conscious sedation<br>☐Deep sedation/monitored anesthesia care (MAC)<br>☐General endotracheal anesthesia<br>☐No sedation<br>☐Unknown
If performed, did the patient experience an adverse cardiopulmonary event related to any of the endoscopic procedures? (select all that apply)	☐ Yes – Mild to moderate respiratory compromise<br>☐ Yes – Severe respiratory compromise<br>☐ Yes – Congestive heart failure/cardiomyopathy<br>☐Yes – Myocardial infarction<br>☐No<br>☐Unknown
Endoscopic findings (please list abnormal findings in impression section of endoscopy report(s))	
Histologic findings (please list abnormal findings in impression section of pathology report(s))	

**Laboratory data and other hepatologic considerations**	
Prior to COVID-19, ideally when patient was healthy:	

© 2021 Yang JY et al. *JAMA Network Open*.

White blood cells (WBC)<br>Hemoglobin<br>Platelets<br>Aspartate aminotransferase (AST)<br>Alanine aminotransferase (ALT)<br>Alkaline phosphatase (ALK-phos)<br>Total bilirubin<br>International normalized ratio (INR)<br>Albumin<br>Factor V level<br>Lipase<br>Creatinine	
At time of hospital admission:<br>White blood cells (WBC)<br>Hemoglobin<br>Platelets<br>Aspartate aminotransferase (AST)<br>Alanine aminotransferase (ALT)<br>Alkaline phosphatase (ALK-phos)<br>Gamma-Glutamyl Transferase (highest) U/L<br>Total bilirubin<br>Direct bilirubin<br>International normalized ratio (INR)<br>Albumin<br>Factor V level<br>Lipase<br>- What is the upper limit of normal<br>Amylase<br>- What is the upper limit of normal<br>Creatinine	
Highest or lowest level during illness:<br>WBC (highest and lowest)<br>Hemoglobin (lowest)<br>Platelets (lowest)<br>AST (highest)<br>ALT (highest)<br>ALK-phos (highest)<br>Gamma-Glutamyl Transferase (highest) U/L<br>Total Bilirubin (highest)<br>Direct Bilirubin (highest)<br>INR (highest)<br>Albumin (lowest)<br>Factor 5 (lowest)<br>Lipase (highest)	

© 2021 Yang JY et al. *JAMA Network Open*.

- What is the upper limit of normal<br>Amylase (highest)<br>- What is the upper limit of normal<br>Creatinine (highest)<br>Absolute lymphocyte count (lowest)<br>C-Reactive Protein (CRP) (highest)<br>Procalcitonin (highest)<br>Troponin (highest)<br>Ferritin (highest) ng/mL or ug/L<br>Interleukin-6 (highest) pg/mL	
Duration between first onset of symptoms and highest AST (days)	
Duration between first onset of symptoms and highest ALT (days)	
Duration between first onset of symptoms and highest total bilirubin (days)	
Duration between first hospital day and highest AST (days)	
Duration between first hospital day and highest ALT (days)	
Duration between first hospital day and highest total bilirubin (days)	
Abnormal LFTs were	☐Still close to max at discharge/death<br>☐Improved but not resolved at discharge/death<br>☐Resolved or close to resolved at discharge/death<br>☐Not applicable
Were the increased LFTs suspected to be due to a drug reaction (based on review of progress/consult notes)?	☐Yes<br>☐No<br>☐Unclear based on records
If yes, which medication(s) were suspected	(medication, dose, route, duration)
If yes, was this a COVID-specific treatment that was believed to increase LFTs (based on review of progress/consult notes)?	☐Yes<br>☐No<br>☐Unclear based on records
Anti-HAV IgM	☐Positive<br>☐Negative<br>☐Not checked
Anti-HCV	☐Positive<br>☐Negative<br>☐Not checked
HCV RNA	☐Positive<br>-Level (in IU/L)<br>☐Negative<br>☐Not checked
HBsAg	☐Positive

	☐Negative<br>☐Not checked
Anti-HBc	☐Positive<br>☐Negative<br>☐Not checked
Anti-HBc IgM	☐Positive<br>☐Negative<br>☐Not checked
Anti-HBs	☐Positive<br>☐Negative<br>☐Not checked
Epstein-Barr Virus antibody IgM (Anti-EBV IgM)	☐Positive<br>☐Negative<br>☐Not checked
Cytomegalovirus antibody IgM (Anti-CMV IgM)	☐Positive<br>☐Negative<br>☐Not checked
Anti-nuclear antibody (ANA)	☐Positive<br>-Level (titer)<br>☐Negative<br>☐Not checked
Anti-smooth muscle antibody (ASMA)	☐Positive<br>☐Negative<br>☐Not checked
Anti-mitochondrial antibody (AMA)	☐Positive<br>☐Negative<br>☐Not checked
Did the patient develop evidence of decompensated liver disease during or shortly after COVID-19 illness? (select all that apply)	☐Yes – hepatic encephalopathy<br>☐Yes – ascites<br>☐Yes – variceal hemorrhage<br>☐Yes – hepatorenal syndrome<br>☐No
Was a liver biopsy performed during the COVID-19 hospitalization?	☐Yes<br>☐No
If yes, histologic findings (please list abnormal findings in impression section of pathology report(s)).	

© 2021 Yang JY et al. *JAMA Network Open*.

eTable. Covariates Used to Calculate Propensity Scores

Covariates
Sex female
Race<br>White<br>Black/ African American<br>Other / Unknown
Ethnicity<br>Hispanic/ Latin(x)<br>Not Hispanic/ Latin(x)<br>Unknown
History of cigarette smoking<br>Current smoker<br>Ex-smoker<br>Non-smoker<br>Unknown
Alcohol use<br>Current<br>Prior<br>None<br>Unknown
Obesity
Diabetes mellitus
Hypertension
Cardiac disease
Pulmonary disease
Active/current malignancy
Immunocompromised status
Luminal gastrointestinal disease
Pancreaticobiliary disease
Chronic liver disease
Presenting symptoms<br>Fever<br>Cough<br>Dyspnea<br>Fatigue/ subjective weakness<br>Myalgia<br>Diarrhea<br>Nausea<br>Vomiting<br>Abdominal pain
COVID-19 treatments<br>Hydroxychloroquine/chloroquine<br>Remdesivir<br>Convalescent plasma<br>Glucocorticoids<br>Tocilizumab
